# Efficacy and neural mechanism of acupuncture for essential hypertension: Study protocol for a randomized clinical trial

**DOI:** 10.1371/journal.pone.0332268

**Published:** 2025-09-19

**Authors:** Xiao-Ya Wei, Xin-Yuan Jiang, Lu Wang, Chao-Qun Yan, Na-Na Yang, Ze-Yi Wang, Xiao Wang, Hang Zhou, Chih-Kai Lee, Cun-Zhi Liu, Xu Wang, Jian-Feng Tu

**Affiliations:** 1 International Acupuncture and Moxibustion Innovation Institute, School of Acupuncture-Moxibustion and Tuina, Beijing University of Chinese Medicine, Beijing, China; 2 School of Life Sciences, Beijing University of Chinese Medicine, Beijing, China; 3 Dongzhimen Hospital, Beijing University of Chinese Medicine, Beijing, China; Alexandria University Faculty of Nursing, EGYPT

## Abstract

**Background:**

Hypertension is a major risk factor for cardiovascular diseases. Acupuncture has been used to control blood pressure in clinical, however, the high-quality clinical evidence is insufficient and the underlying mechanisms remain unclear.

**Objectives:**

This study aims to evaluate the efficacy of electroacupuncture for patients with essential hypertension (EH) compared with sham electroacupuncture and explore the neural mechanism.

**Study design and methods:**

his is an 8-week, parallel-grouped, randomized clinical trial. A total of 66 patients diagnosed with EH are randomly assigned (1:1) to receive either the electroacupuncture or sham electroacupuncture treatment 3 sessions per week for 4 weeks. The primary outcome is the mean systolic blood pressure change at week 4 from the baseline measurement. The independent sample t-test will be used to compare the differences between two groups. The secondary outcomes include the change in mean diastolic blood pressure, the proportion of patients with controlled blood pressure (< 140/90 mm Hg), and quality of life. The outcome variables are measured at baseline, week 2, week 4 and week 8. Functional magnetic resonance imaging scans, pulse rate variability acquisition, and image-based photoplethysmography recording are performed at baseline and the end of the intervention period to explore acupuncture-related neuroplasticity changes. Correlation analyses are performed to investigate the relationships between the neural changes and clinical symptoms. Adverse events during the trial are monitored.

**Trial registration:**

Chinese Clinical Trials Registry, the number is: ChiCTR2400082315 (https://www.chictr.org.cn/showproj.html?proj=223840).

## Background

Hypertension is a common risk factor for cardiovascular diseases, accompanied by increased blood pressure and autonomic dysfunction, which can cause a wide range of damage to the brain, heart, and blood vessels [1,2]. It is estimated that more than 30% of adults worldwide suffer from hypertension, with a huge economic burden on society [3,4]. Long-term elevation of blood pressure could exacerbate brain degeneration in hypertensive patients, leading to an increase in the risk of cardiovascular events [5,6]. Surveys of blood pressure-related diseases show that about 13.5% of global premature deaths (about 7.6 million people) are attributable to hypertension [7]. However, the awareness and control of hypertension remain insufficient in many countries [8], and 87% population of the nearly 300 million patients with hypertension in China have poorly controlled blood pressure [9].

Antihypertensive drugs are often used to hypertension treatment, but existing some adverse effects and low adherence [10]. The guidelines recommend lifestyle modifications also as first-line therapy for hypertension patients, but are difficult to maintain in the long-term [11]. Therefore, it is important to seek effective adjuvant treatment of hypertension. Acupuncture has been used as a non-pharmacological treatment for cardiovascular diseases [12,13], and some studies suggest that acupuncture could lower blood pressure [14–16]. However, a Cochrane review showed that the efficacy of acupuncture on the reduction of blood pressure is still not widely recognized due to insufficient high-quality clinical evidence [17].

It is suggested that the imbalance in the autonomic nervous system with enhanced sympathetic activity is one of the pathogenesis of hypertension, which is controlled by key brain nuclei and neural circuits in the central nervous system [18,19]. Studies have found that the regulation of autonomic parameters is associated with lower blood pressure in hypertensive people [20]. However, whether acupuncture regulates blood pressure by affecting the autonomic nervous system and the central mechanism is unclear. Functional magnetic resonance imaging (fMRI) could observe the functional activity changes of the brain, and in recent years, it has been used to explore the central mechanism of acupuncture effect [21]. In addition, contact and image-based photoplethysmography (PPG) could detect important body physiological and pathological information (e.g., pulse rate variability [PRV] and blood pressure) from the transformed pulse wave signal to detect autonomic neuromodulation, and they also could be used as the non-invasive quantitative indicators of sympathetic and parasympathetic tone and functional status in clinical practice [22–24]. Based on the above, we aims to perform a randomized controlled clinical trial in patients with essential hypertension (EH) to evaluate the efficacy and safety of electroacupuncture (EA) vs. sham electroacupuncture (SE). The second objective is to preliminarily assess the underlying central integration mechanism of acupuncture on reducing blood pressure by combining neuroimaging and neural detection techniques.

## Methods and design

### Study design

**Fig 1:**
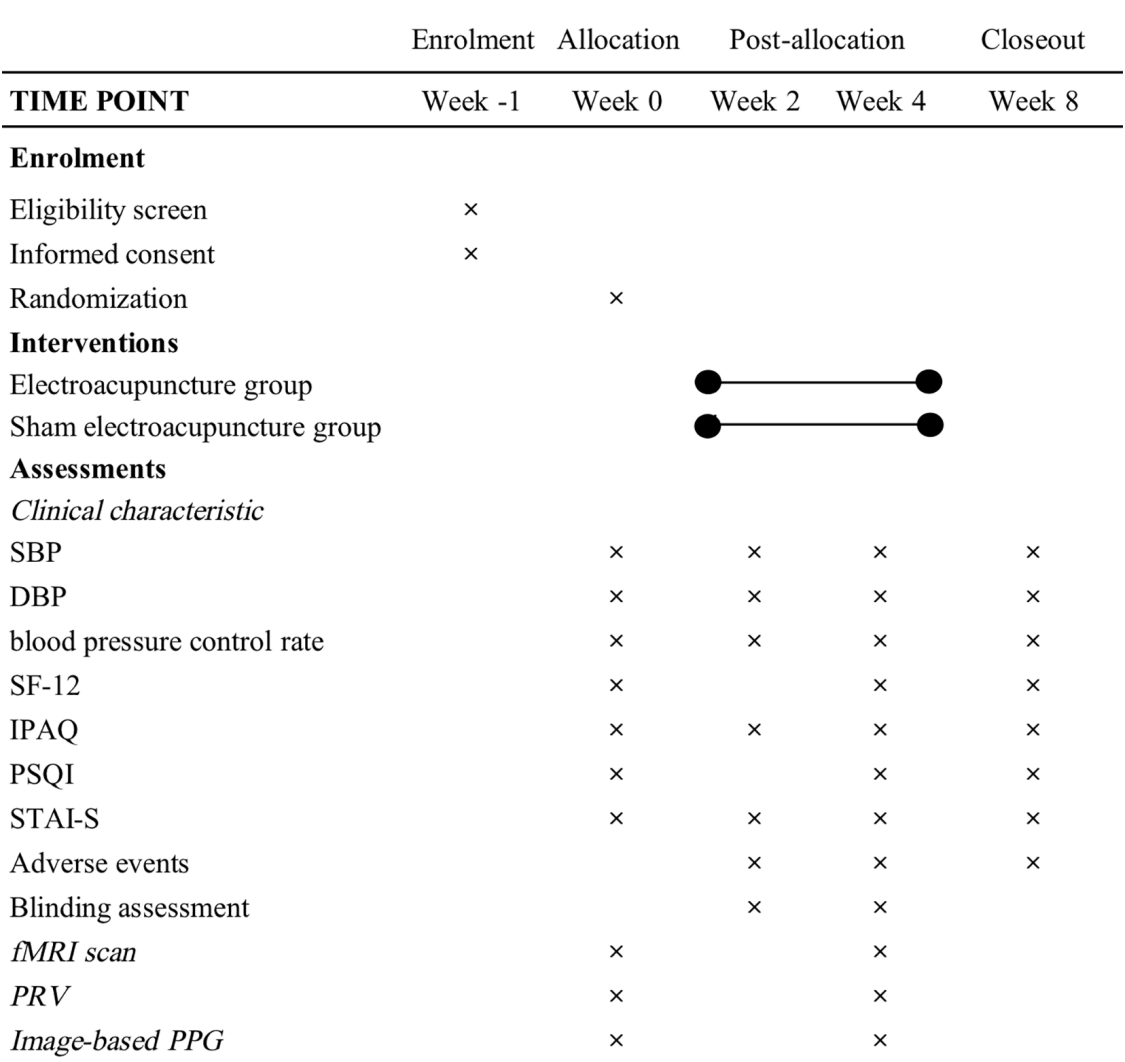
Schedule of enrollment, intervention, and assessment. Schedule of enrollment, intervention, and assessment. SBP, systolic blood pressure; DBP, diastolic blood pressure; SF-12, 12-item Short Form Health Survey; IPAQ, International Physical Activity Questionnaire; PSQI, Pittsburgh Sleep Quality Index; STAI-S, State-Trait Anxiety Scale-State Anxiety Subscale; fMRI, functional magnetic resonance imaging; PRV, pulse rate variability; PPG, photoplethysmography.

**Fig 2:**
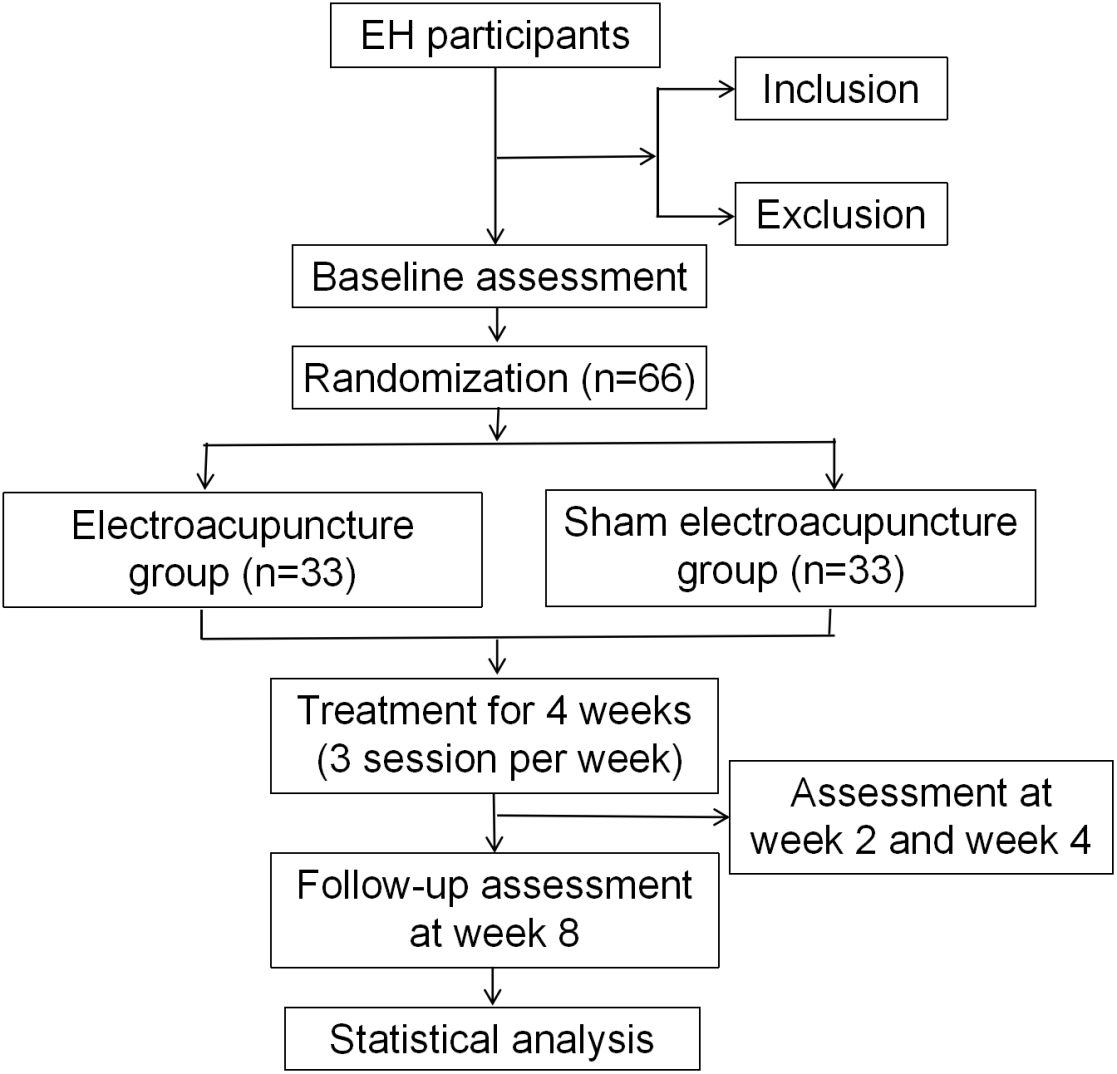
Flow diagram. EH, essential hypertension.

This is a randomized controlled, parallel trial. A total of 66 participants will be recruited for the study, with an average of 33 in each group. The protocol will be reported following Standard Protocol Items: Recommendations for Interventional Trials (SPIRIT) schedule statement (S1 File) and the Standards for Reporting Interventions in Clinical Trials of Acupuncture (STRICTA) guidelines (S2 File). The schedule of enrolment, intervention, and assessments is shown in Fig 1. The procedure of the trial is shown in Fig 2.

### Study setting and recruitment

The study will be carried out at the Dongzhimen Hospital Affiliated to the Beijing University of Chinese Medicine. The study will recruit participants with EH who meet the diagnostic criteria according to the European guideline [25] and Chinese guideline [26] through hospital outpatient. Meanwhile, the recruitment of participants starts in September 2024. This study recruitment period is expected between September 2024 and October 2025. The trial is planned to be completed in December 2025. Additionally, 33 healthy controls (HCs) will be recruited through social media advertisements.

### Participants

#### Inclusion criteria.

1. Meeting the diagnostic criteria for EH, according to the European and Chinese guidelines for hypertension.
2. Office systolic blood pressure (SBP) from 140 to 159 mm Hg and diastolic blood pressure (DBP) from 90 to 99 mm Hg.
3. Not taking any antihypertensive medication.
4. Aged 25–60 years, male or female, right-handedness.
5. No language or intellectual disabilities for answering and completing the questionnaires.
6. Willing to sign the informed consent.

#### Exclusion criteria.

1. Other diseases that can affect the blood pressure, including but not limited to diseases of renal parenchyma, renal artery stenosis, obstructive sleep apnoea syndrome, and primary hyperaldosteronism.
2. Receiving medicines that affect blood pressure except antihypertensive drugs in last month, including but not limited to glucocorticoids and central nervous system inhibitors.
3. Uncontrolled diabetes (HbA1c≥6.5%).
4. Drug or alcohol abuse.
5. Pregnant and lactating women.
6. Having fMRI contraindications, such as pacemakers, defibrillators, vascular clips, implanted electrical or magnetic devices, mechanical heart valves, cochlear implants, and claustrophobia; engaged in metal related work with metal fragments in the face or eyes; severe skull anatomical asymmetry or definite lesions were found in MRI scanning.
7. Received acupuncture treatment for hypertension within 3 months.
8. Participated in other clinical trials in the past 1 months.

### Informed consent and participant safety

Patients will be asked to sign an informed consent form prior to enrollment (provided in the S3 File). During the trial, the researchers will monitor and record adverse events and reactions throughout the acupuncture treatment, such as panic, subcutaneous hematoma, and pain. If the patient is unable to tolerate these adverse effects, they will be allowed to withdraw from the study.

### Randomization

In this study, EH patients will be randomly divided into an EA group and a SE group in a ratio of 1:1, with 33 participants in each group. The random sequence will be generated by a independent statistician (not involved in the implementation or statistical analysis) with the software Stata V.12.0 using block randomization, with a block size varying randomly between 4 and 6. Allocation concealment is implemented by a dedicated random number administrator. The random numbers will be kept by a same personnel who is not involved in the treatment and clinical measurement of this study. After the baseline assessment of eligible patients, a random number and group will be told to the acupuncturist by this fixed person via a mobile phone message.

### Blinding

Acupuncturists cannot be blind, but they are asked not to inform patients about treatment options, maintain neutral communication and will not participate in outcome assessments. We provide unified training for acupuncturists. To reduce potential bias, patients, data collectors and statistician (involved in data analysis) will be blinded to the group until the trial is completed. After treatment, the blinding assessment will be conducted on the participants, asking them the question, ‘Which type of acupuncture do you think you received, EA or Microelectroacupuncture or Unclear?’. The James blinding index (range 0–1) will be used to assess the blinding (0, total absence of blinding; 1, complete blinding; 0.5, completely random blinding).

### Sample size

The sample size is calculated based on the decrease in SBP from baseline to week 4. According to the prior research [27–30], we assume that the mean SBP change value at week 4 in the EA group reaches 5 mm Hg with a standard deviation of 5 mm Hg, and in the SE group, the mean SBP change value reaches 1 mm Hg with a standard deviation of 5 mm Hg. The sample size of 26 patients in each group was calculated to provide 80% power, with a two-tailed α level of 0.05, using PASS 2021 software. This requires 33 patients per group allowing for 20% dropout.

### Interventions

#### Electroacupuncture.

**Fig 3:**
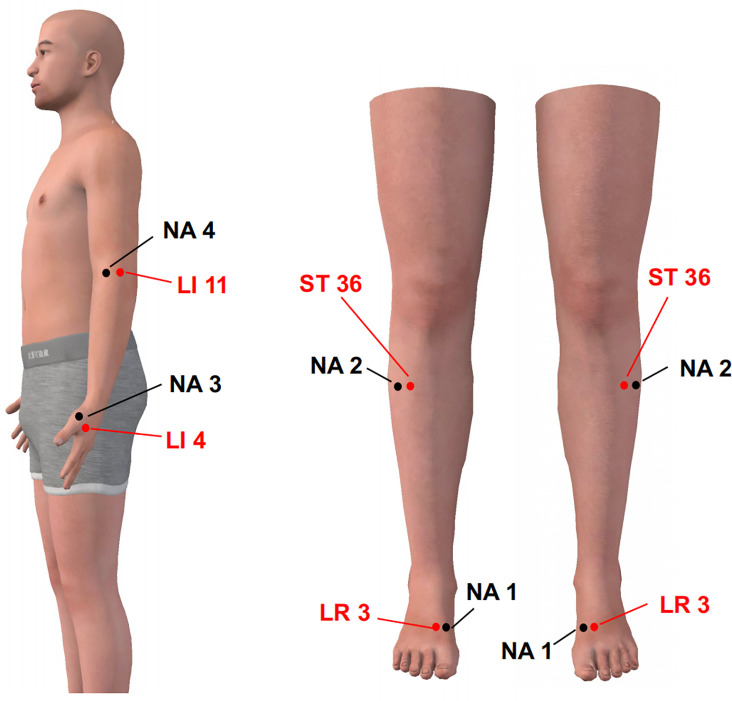
Locations of bilaterally acupoints and non-acupoints. LI, large intestine meridian; LR, liver meridian; ST, stomach meridian;NA, non-acupoint.

Patients will receive 12 sessions of electroacupuncture treatment (3 sessions weekly for 4 weeks). The acupoints will include bilateral *Hegu* (LI4), *Quchi* (LI11), *Zusanli* (ST36), and *Taichong* (LR3) (Table 1 and Fig 3), according to WHO Standard Acupuncture Locations. These acupoints are selected based on previous research and existing evidence, their combined use has a synergistic effect in lowering blood pressure [27,31]. Another multicenter clinical trial of acupuncture for hypertension also supported the clinical efficacy of the combined use of these acupoints [32]. Furthermore, the mechanism reseach suggested that electroacupuncture stimulation of Taichong and Quchi could effectively reduce the blood pressure and the excitability of sympathetic nerve in the hypothalamic paraventricular nucleus (PVN) in spontaneous hypertensive rats [33]. Electroacupuncture stimulation of Zusanli might affect blood pressure by regulating the function ofγ-aminobutyric acid receptors in the nucleus solitularis [34]. Acupuncture intervention at the Taichong could reduce the blood pressure of spontaneously hypertensive rats and inhibit the activity of nicotinamide adenine dinucleotide phosphate oxidase in the lateral ventral medulla oblongata of their heads [35].

**Table 1:** Locations of acupoints for electroacupuncture group.

Acupoints	Locations
Taichong (LR3)	In the depression anterior to the junction of first and second metatarsal bones.
Zusanli (ST36)	3 cun* directly below Dubi (ST35), and one finger-breadth lateral to the anterior border of the tibia.
Hegu (LI4)	On the dorsum of the hand, radial to the midpoint of the second metacarpal bone.
Quchi (LI11)	On the lateral aspect of the elbow, at the midpoint of the line connecting LU5 with the lateral epicondyle of the humerus.

*1 cun (≈20 mm) is defined as the width of the interphalangeal joint of patient’s thumb.

**Fig 4:**
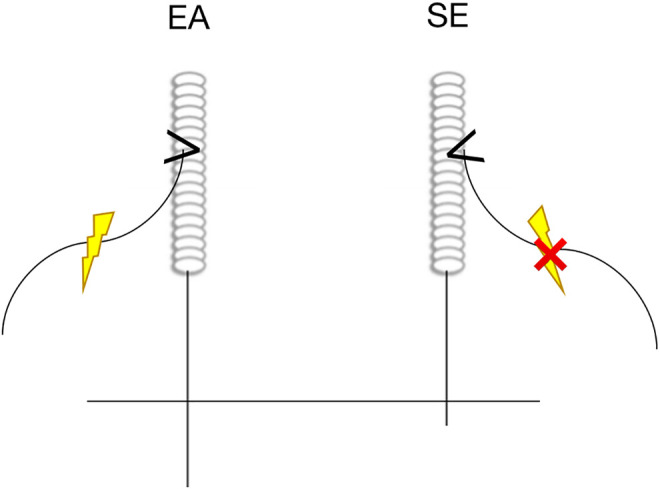
The operational diagram of the intervention. EA, electroacupuncture; SE, sham electroacupuncture.

Before needle insertion, both the acupuncturist’s hands and acupuncture site will be strictly disinfected with 75% alcohol. Then, single-use acupuncture needles (0.30 * 40 mm, Hwato, Suzhou, China) will be inserted vertically into acupoints, and acupuncturists will manually stimulate the needles (twirling, lifting, and thrusting) to achieve ‘de qi’ sensation (soreness, numbness, distension or heaviness). For each treatment, paired electrodes from the Hwato SDZ-II EA apparatus (Suzhou, Jiangsu Province, China) will be attached to needle holders at LI 4–LI 11 and ST 36–LR 3 acupoints on one side of the body. Attach the EA apparatus to the opposite needle holders at the next treatment. The EA stimulation will be a dilatation wave of 2 Hz, depending on the patient’s comfort level (Fig 4). The treatment will be left for 30 min.

#### Sham electroacupuncture.

Four non-acupoints are preset for sham electroacupuncture treatment. The location of the non-acupoints is shown in Table 2 and Fig 3. Acupuncture is a treatment method that stimulates specific acupoints along the meridians on the body surface. These non-acupoints locations are areas near or around the actual acupoints and without therapeutic effect. The acupuncture needles and disinfection procedures will be the same as the electroacupuncture group. The acupuncture needles will be inserted into the skin 4 mm without manipulations or ‘de qi’ sensation. Paired electrodes from the Hwato SDZ-II EA apparatus (Suzhou, Jiangsu Province, China) will be attached to needle holders at NA 1–NA 2 and NA 3–NA 4 points on one side of the body without electric current output. At the next treatment, the sham EA apparatus will be attached to the opposite needle holders (Fig 4). This shallow puncture method could avoid the introduction of confounding factors (such as skin contact effects) due to different stimulation methods, and increase the success rate of blinding for patients (penetrating operations are closer to the real treatment experience). The duration of the treatment will be left for 30 min. The patient will receive 12 sessions of EA treatment (3 times a week for 4 weeks).

**Table 2:** Locations of non-acupoints for sham electroacupuncture group.

NA	Locations
NA1	1 cun beside LR3, on the first metatarsal bone.
NA2	1 cun back and outside of ST36.
NA3	In the middle of LI4 and LU10 (between the lung and large intestinal meridian).
NA4	In the middle of LI11 and LU5 (between the lung and large intestinal meridian).

NA non-acupoints.

*1 cun (≈20 mm) is defined as the width of the interphalangeal joint of patient’s thumb.

All the above treatments were carried out by licensed acupuncturists with at least three years of acupuncture experience. The acupuncturist will keep a record of each treatment. During the trial, patients will maintain the same lifestyle as before. If the patient’s blood pressure is poorly controlled, we will consult with a professional doctor to help the patient develop an effective antihypertensive therapy. Medication regimens will be recorded. The HCs with normal blood pressure will not receive any intervention.

### Clinical outcome assessments

The primary outcome will be the mean difference in the changes in office SBP from baseline to 4 weeks between the two groups. SBP will be measured using a unified automated monitor (HEM-7136, Omron, Kyoto, Japan), and follow the WHO STEPS protocol for the procedure. The arm with higher blood pressure in the patient at baseline will be selected as the measurement arm throughout the study. Before each measurement of the patient’s blood pressure, the patient will be asked to sit and rest for 5 minutes with the upper arm at the level of the heart. Then, the blood pressure measurement will record three values in succession at 5-minute intervals, and the final blood pressure being the average of the last two times [36,37].

Secondary outcomes will include differences in the changes in office DBP between the two groups; the proportion of patients with well-controlled blood pressure (office blood pressure <140/90 mm Hg); and differences in the changes in the quality of life assessed using 12-item Short Form Health Survey (SF-12) [38]; physical activity assessed using International Physical Activity Questionnaire (IPAQ) [39]; quality of sleep assessed using Pittsburgh Sleep Quality Index (PSQI) [40]; the severity of current anxiety symptoms assessed using State-Trait Anxiety Scale State Anxiety Subscale (STAI-S) [41].

Safety outcomes will be any acupuncture-related adverse events (pain, panic, and subcutaneous hematoma) at weeks 2, 4, and 8 in both groups. All patients will be asked to guess which treatment they have received at week 2 and week 4 as blinding assessments. All patients’ physical condition, cognitive function, or other eligibility will be monitored during the trial.

### MRI data acquisition

The MRI scan will be performed with a 3.0 Tesla scanner (Siemens, Germany) in the Dongzhimen Hospital Affiliated to the Beijing University of Chinese Medicine. Patients will undergo fMRI scans at baseline and the end of the intervention. In addition, the HCs will undergo only one scan. Before scanning, they will be required to wear earplugs and remove all magnetic and metal items. In the meantime, they will be asked to maintain a comfortable supine position and reduce head movements.

Echo planar imaging (EPI) sequence is used for resting-state fMRI scanning: whole brain, repetition time (TR) = 2000 ms, echo time (TE) = 30 ms, field of view (FOV) = 224 mm × 224 mm, flip angle (FA) = 90°, slice thickness = 3.5 mm, axial slices = 32, size of voxel = 3.5 mm × 3.5 mm × 3.5 mm, in-plane resolution = 64 × 64, and volumes = 240.

### Photoplethysmography assessment

Pulse oximetry collects pulse waves from fingertips by PPG measurements for PRV analysis. Image-based PPG that could measure skin blood flow fluctuations from raw digital camera images and allow us to extract pulse wave signals from video. In this study, we will use a finger-clip pulse oximeter (CONTCS, CMS60E) to collect the pulse signal for 2 minutes. In addition, we will use a smartphone camera (iPhone 14 Pro) to record a video (40 seconds) of the acupuncture points in the arm at a speed of 30 frames per second with a resolution of 1920 *1080 pixels. All patients will be evaluated before and at the end of treatment, respectively. Autonomic activity could also be reflected by analyzing the morphology of signal waves. In this study, we will mainly analyze the general time domain parameters and frequency domain parameters.

### Data management and quality control

Clinical data for all patients will be collected using a case report form (CRF). All researchers will be trained in questionnaire evaluation and data management. The data will be entered into an Excel spreadsheet independently by the two researchers on time, and then the data will be checked for completeness and accuracy. When there are discrepancies, refer to the original CRF to correct the data. All paper and electronic documents in this study will be reasonably kept. If readers and reviewers have any questions regarding our published data, they can contact the corresponding author to request the original data. In addition, we will protect patients’ private information, such as names and phone numbers. The patients will be scanned by a professional imaging technician on the same MRI scanner and the quality of the imaging data will be checked after each scan. Finally, we will establish a trial steering committee composed of the primary investigators (LCZ, TJF and WX) and statistician to monitor the progress and data quality of this study every 3 months.

### Statistical analysis

#### Clinical data analysis.

The clinical data will be analyzed by an independent statistician using IBM SPSS V.20.0 software, and the *P* values < 0.05 will be considered statistically significant. For those conforming to a normal distribution, the data will be described utilizing the mean (standard deviation), otherwise, the median (quartile spacing) will be used. The categorical data will be described as percentages (%). We will use the independent sample t test to compare the effect of EA vs. SE on blood pressure from baseline to week 4. For the other outcomes, if data conforms to a normal distribution, it will undergo t-test analysis. Conversely, if it does not conform to the normal distribution, it will be analyzed by nonparametric test. Categorical data will be analyzed via the χ^2^ test or Fisher’s exact test. All cases of randomized patients will be undertaken on the intention-to-treat analysis, and missing values will be filled in using multiple imputations. To evaluate the stability of results, we will conduct a sensitivity analysis with the last observation values. A per-protocol analysis will include the patients who complete at least 80% of the study.

#### Neuroimaging data analysis.

The fMRI data will be analyzed using the Data Processing Assistant and Resting-State fMRI (DPARSF) and SPM toolbox based on MATLAB software (Mathworks, Inc., Natick, MA, USA). The preprocessing will include DICOM format conversion, removing the first 10 volumes, slice timing, head motion correction, spatial standardization (resampled to 3 mm × 3 mm × 3 mm), removing linear trend, low-frequency filtering (0.01–0.08 Hz), and regression removal of covariates (head motion parameters, white matter signal, grey matter signal or/and global signal), spatial smoothing with 6 mm FWHM Gaussian kernel. Participants with more than 3 mm of displacement in x, y, or z or 3 degrees of angular head motion will be excluded in further analyses. After data preprocessing, we will compare the brain region changes between EH patients and HCs. Then the amplitude of low-frequency fluctuation (ALFF), regional homogeneity (ReHo), or functional connectivity (FC) analysis will be performed to investigate the brain responses significantly associated with the antihypertensive effects of acupuncture. Gaussian Random Field (GRF) correction will be applied for multiple comparison correction. These indicators are the most commonly used in current fMRI analysis. Previous studies found that there are abnormal ReHo values of insula and enhanced FCs between brain networks in the hypertension patients [42,43]. Pearson or Spearman correlation analysis will be used to test the correlation between fMRI data and clinical measurements. The False Discovery Rate (‌FDR) correction is applied to correct the potential type I errors caused by multiple testing. Then, we will further conduct multiple regression and support vector regression (SVR) analyses to explore the predictive factors affecting the therapeutic effect of acupuncture for blood pressure.

### Patient and public involvement

The corresponding author (JFT) is a patient with hypertension. He was involved in the design and conduct of the trial. There is no other public involvement in the study design, recruitment, or conduct of the study.

### Ethics and dissemination

This protocol is guided by the principles of the Helsinki declaration. The study has been approved by the Medical Ethical Committee of Beijing University of Chinese Medicine (No. 2024BZYLL0107; S4-S6 Files) and registered on Chinese Clinical Trials Registry (ChiCTR2400082315). Patients can decide to withdraw from the trial at any time, and all their information will be kept confidential. The results will be disseminated through publishing in peer-reviewed academic journals. Any part of the research or preliminary data has not been submitted or published elsewhere at present.

## Discussion

Acupuncture could lower blood pressure and is used as a non-pharmacological treatment for the treatment of cardiovascular disease as well as for the relief of clinical symptoms. However, the effects of acupuncture on the cardiovascular and nervous systems remain unclear. In this study, we design a randomized clinical trial to evaluate the efficacy and safety of EA vs. SE for lowering blood pressure in patients with EH. We will recruit 66 eligible patients for 12 treatments over 4 weeks, with blood pressure measurements and scales before and after treatment. At the same time, we measure and analyze neuroplasticity changes related to treatment outcomes through neuroimaging. In addition, we evaluate the relationship between acupuncture treatment for EH in clinical variables and altered neural representations. The results of this study may have important clinical and research implications.

Currently, there is little high-quality clinical evidence on the role of acupuncture in blood pressure management. Acupuncture has been reported to have no benefit in lowering blood pressure, possibly due to the low methodological quality of clinical trials, the lack of blinding and safety assessment, as well as problems in the setting of the control group [17,44]. Based on the above summary, in this study, a strict randomized controlled trial design is adopted, sham electroacupuncture is set as the control group, and the blinding and adverse event evaluations are conducted. In addition, our understanding of the biological basis of the cardiovascular and neuromodulator aspects of acupuncture is limited. Macefield et al. proposed that the human sympathetic connectome of the insula-hypothalamus-rostral ventrolateral medulla circuit is a key component of the central autonomic network at the brainstem level and plays an important role in regulating blood pressure [45], which is also consistent with the data obtained from experimental animals [46,47]. Neuroimaging studies in spontaneously hypertensive rats have found that acupuncture could regulate blood pressure by influencing the functional brain regions such as the hypothalamus and insula [48,49]. However, few studies have evaluated changes of the functional activity in these brain regions of the neural circuit after treatment in patients with hypertension. This study introduces neuroimaging techniques into a rigorous randomized controlled trial of acupuncture for the treatment of EH. Combining contact and image-based PPG data, this study will provide important multi-modal data to understand the integrative mechanism of acupuncture on blood pressure and autonomic regulation.

Our study has several potential limitations. First, this is a single-center study, which may be limited in extrapolation and representativeness, however, the quality of this trial is more easily controlled compared to multicenter trials. In addition, the patients included in this trial are concentrated at 25–60 years old, and the findings should be further validated and explored in the elderly and young populations. Third, this study lacks long-term follow-up. Future research will extend the follow-up to 12–24 weeks to evaluate the durability of acupuncture for hypertension over a more longer period of time.

## Conclusions

In conclusion, this trial will provide important clinical support for the efficacy and safety of acupuncture for EH, and preliminarily explore the underlying neural mechanism, which may provide precise evidence for non-pharmacological blood pressure management.

## Supporting information

S1 File SPIRIT checklist. (PDF)

S2 File STRICTA 2010 checklist. (PDF)

S3 File Informed consent form. (DOC)

S4 File Original protocol approved by the ethics committee. (DOCX)

S5 File Original protocol approved by the ethics committee (Chinese). (DOCX)

S6 File Ethical approval document. (DOCX)

## Abbreviations

EH: Essential hypertension
fMRI: Functional magnetic resonance imaging
PPG: photoplethysmography
PRV: Pulse rate variability
EA: Electroacupuncture
SE: Sham electroacupuncture
HCs: Healthy controls
SBP: Systolic blood pressure
DBP: Diastolic blood pressure
SF-12: 12-item Short Form Health Survey
IPAQ: International Physical Activity Questionnaire
PSQI: Pittsburgh Sleep Quality Index
STAI-S: State-Trait Anxiety Scale State Anxiety Subscale
CRF: Case report form
