# Supplementary material for: Efficacy and neural mechanism of acupuncture for essential hypertension: Study protocol for a randomized clinical trial
Source: PLoS One. 2025 Sep 19;20(9):e0332268. doi: 10.1371/journal.pone.0332268 (PMC12449014; doi:10.1371/journal.pone.0332268)
Supplement: S2 File — (PDF) [file pone.0332268.s002.pdf]

STRICTA 2010 checklist of information to include when reporting interventions in a clinical trial of acupuncture

Item	Detail	Reported on page No
**1.Acupuncture rationale**	1a)Style of acupuncture (e.g.Traditional Chinese Medicine,Japanese,Korean,Western medical,Five Element,ear acupuncture,etc)	page 3
	1b)Reasoning for treatment provided,based on historical context,literature sources,and/or consensus methods,with referenceswhere appropriate	page 3
	1c)Extent to which treatment was varied	page 3
**2.Details of needling**	2a)Number of needle insertions per subject per session (mean and range where relevant)	page 7-9
	2b)Names(or location if no standard name)of points used (uni/bilateral)	page 7-9
	2c)Depth of insertion,based on a specified unit of measurement,or on a particular tissue level	page 7-9
	2d)Response sought(e.g.de qi or muscle twitch response)	page 7-9
	2e)Needle stimulation (e.g.manual,electrical)	page 7-9
	2f)Needle retention time	page 7-9
	2g)Needle type(diameter,length,and manufacturer or material)	page 7-9
**3.Treatment regimen**	3a)Number of treatment sessions	page 7-9

	3b)Frequency and duration of treatment sessions	page 7-9
**4.Other components of treatment**	4a)Details of other interventions administered to the acupuncture group(e.g.moxibustion,cupping,herbs,exercises,lifestyle advice)	page 10
	4b)Setting and context of treatment,including instructions to practitioners,and information and explanations to patients	page 10
**5.Practitioner background**	5)Description of participating acupuncturists (qualification or professional affiliation,years in acupuncture practice,other relevant experience)	page 10
**6.Control or comparator interventions**	6a)Rationale for the control or comparator in the context of the research question,with sources that justify this choice	page 9
	6b)Precise description of the control or comparator.If sham acupuncture or any other type of acupuncture-like control is used,provide details as for Items 1 to 3 above.	page 9

Note:This checklist,which should be read in conjunction with the explanations of the STRICTA items provided in the main text,is designed to replace CONSORT 2010's item 5 when reporting an acupuncture trial.
