# Supplementary material for: Efficacy and neural mechanism of acupuncture for essential hypertension: Study protocol for a randomized clinical trial
Source: PLoS One. 2025 Sep 19;20(9):e0332268. doi: 10.1371/journal.pone.0332268 (PMC12449014; doi:10.1371/journal.pone.0332268)
Supplement: S3 File — (DOC) [file pone.0332268.s003.doc]

**Informed consent form· Informed notice page**

Dear Sir/Madam:

You will be invited to participate in a study titled "Neuroimaging study on the central integration mechanism of acupuncture regulation in essential hypertension based on insula- hypothalamus-rVLM." This study aims to evaluate the effectiveness and safety of acupuncture intervention for essential hypertension (EH) and explore the central response mechanisms that produce clinical therapeutic effects. Before you decide whether to agree to participate in this study, please read the following carefully. It will help you understand the study, why it is being conducted, the procedures and duration of the study, as well as the potential benefits, risks, and discomforts that may arise from participating. If you wish, you can also discuss with your family or friends, or ask your doctor for an explanation to assist you in making your decision.

**Ⅰ. Introduction to the research project**

1. Project name, sponsor, writing version number or date

We will conduct a study on the "Neuroimaging study on the central integration mechanism of acupuncture regulation in essential hypertension based on insula- hypothalamus-rVLM." This research is supported by the National Natural Science Foundation of Chinas Key Project (Project Number: No.82330126). The project leader is Professor Liu Cunzhi, Dean of the School of Acupuncture-Moxibustion and Tuina at Beijing University of Chinese Medicine. (Version Number: V5.0 Version Date: March 4, 2025)

2. Research objectives

To verify whether acupuncture for 4 weeks can play a better role in lowering blood pressure and improving quality of life for patients with EH, and explore the central integration mechanism of acupuncture producing clinical efficacy.

1. Study inclusion criteria

(1) Inclusion Criteria: ① Grade 1 hypertension, systolic blood pressure SBP between 140-159 / diastolic blood pressure DBP between 90-99 mmHg; ② Not using antihypertensive drugs; ③ Age 25-60 years, male or female, right-handed; ④ No language or cognitive impairment, able to answer and complete the questionnaire smoothly; ⑤ Signed informed consent form.

(2) Exclusion Criteria: Patients with the following conditions should not participate in this study: ① Renal parenchymal diseases, renal artery stenosis, and other vascular diseases, obstructive sleep apnea syndrome, primary aldosteronism, and other conditions affecting blood pressure; ② Use of other medications that affect blood pressure within the past month (excluding antihypertensive drugs): such as hormonal medications, central nervous system drugs, nonsteroidal anti-inflammatory drugs, etc.; ③ Uncontrolled diabetes (glycated hemoglobin ≥6.5%); ④ Suspected or confirmed history of alcohol or drug abuse; ⑤ Women of childbearing age who are not using contraception, pregnant women, and breastfeeding women; ⑥ Individuals carrying pacemakers, defibrillators, vascular clips, implanted electrical or magnetic devices, mechanical heart valves, cochlear implants, or functional MRI contraindications, and individuals with claustrophobia; those with metal fragments in their face or eyes, or working with metals; individuals found to have clear organic lesions or severe cranial anatomical asymmetry on MRI; individuals unable to undergo fMRI due to other reasons; ⑦ Individuals who have received acupuncture treatment for hypertension within the past 3 months; ⑧ Patients who participated in other clinical trials within the past month.

(3) Exit and exclusion criteria

Dropout Criteria: ① Participants who have provided informed consent and been screened to qualify for the randomized trial but fail to complete the prescribed course of treatment and observation period (≤80% of the specified treatment) are considered dropout cases. ② Handling of Dropout Cases: When a participant drops out, the researcher should contact them by home visit, scheduled follow-up, or phone call, to inquire about the reasons and complete necessary assessments. All dropout cases should have their relevant trial data properly preserved, both for archiving and for statistical analysis in the full dataset. No additional supplementation is required for dropout patients.

Exclusion criteria: ① Those who meet the inclusion and exclusion criteria but cannot cooperate with treatment on time should be excluded. ② After the subject is excluded, the investigator should inquire about the changes of the condition by telephone and record them. There is no need to supplement the excluded patients.

4. Experimental process

First, the researcher asks about your illness and previous treatment. Based on the diagnostic, inclusion and exclusion criteria, determine whether you can participate in this trial. If you are selected for the trial, you will receive acupuncture treatments three times a week for four weeks, totaling 12 sessions. At the end of the eighth week, a dedicated evaluator will call you to schedule an in-person evaluation. If you cannot come in person, the evaluation will be conducted by phone. If there is no answer, follow-up will be made using the address or WeChat ID you provided.

5. The time and duration of participation in the study

This study lasts for 8 weeks. If you participate in this study, the investigator will contact you during this period to complete relevant treatment and evaluation.

6. Grouping situation

If you meet the requirements of this trial, there will be a 50% chance of being assigned to the acupuncture group, and a 50% chance of being assigned to the shallow acupuncture group. You will be assigned to either the shallow needling group or the deep needling group. Both groups will receive 12 treatments for a total of 66 people. If you are eligible for an MRI scan, you will receive two free head MRI scans within one week before treatment and one week after treatment.

The following conditions are not suitable for MRI: working in a metal environment; having claustrophobia; having had Surgery with metal in body; history of head trauma; history of eye injury (involving metal); pregnancy or lactation; history of stroke/fainting.

**Ⅱ. Research unit and personnel qualifications**

The recruitment and treatment sites for this study are set at Beijing University of Chinese Medicine and its affiliated Dongzhimen Hospital. The MRI examination site is designated at Dongzhimen Hospital. Professor Liu Cunzhi, the project leader, has long been engaged in acupuncture research and clinical work. His research focuses on the specific effects of acupoints and their compatibility mechanisms, conducting multicenter clinical trials for conditions such as migraines, knee pain, functional dyspepsia, and dementia. He has led and participated in several major projects, including those under the National 973 Program and the National Natural Science Foundation, accumulating rich clinical and research experience. All acupuncturists involved in the trials must hold a Chinese Medical Practitioner Qualification Certificate issued by the Peoples Republic of China and have more than five years of clinical work experience.

**Ⅲ. Describe the potential benefits of participating in this study**

1. Study the benefits to social groups:

Through acupuncture intervention in this study, it is possible to lower blood pressure and improve the quality of life of participants. We hope that the information we get from your participation in this study will benefit other participants with similar conditions in the future.

2. Study benefits to the subject:

This study provides 12 sessions of acupuncture over 4 weeks that may lower blood pressure and improve your quality of life. However, we cannot guarantee to improve your health.

**Ⅳ. Study the possible discomfort and risk to the subject**

When you decide whether to participate in this study, please consider carefully how the treatment and follow-up will affect your daily work and home possible impacts such as family life. Consider the timing of each treatment and follow-up with transportation issues if you are involved in the trial. If you have any questions about the contents, please consult us. For your safety and to ensure the validity of the study results, you may not participate in any other clinical studies relating to acupuncture, drugs and medical devices during the study period.

There is a risk of adverse reactions during the trial. A large number of clinical studies on acupuncture and moxibustion have been carried out in the past. The minor adverse events that may occur are mainly skin bruising, needle fainting, needle stagnation, and acid distension after acupuncture treatment. If the above situation occurs, we will suspend acupuncture treatment and decide whether to continue treatment after the adverse reactions are alleviated.

A large number of imaging clinical studies have shown that there are no serious adverse reactions from the imaging studies at present. However, participants may experience claustrophobia or other adverse reactions after entering the MRI machine. If any of these situations occur, we will immediately stop the MRI scan, guide the participant out of the MRI room, and promptly consult with the relevant specialists for further evaluation and handling.

**V. Emergency plan that can be adopted when an emergency occurs during the trial treatment**

If you experience any adverse reaction or discomfort during the trial; or if the interval is>3 days, SBP > 20 mmHg or DBP > 10 mmHg, or if your SBP increases by more than 20 mmHg or your DBP increases by more than 10 mmHg compared to before, you should immediately report this to your research doctor. This is crucial. If you or your research doctor determines that you cannot tolerate these adverse reactions, the intervention may involve completely discontinuing the treatment, and you might be asked to withdraw from the study. If the blood pressure control is poor, a plan for managing blood pressure will be determined based on your condition, such as adjusting lifestyle habits or appropriately increasing antihypertensive medication. If you experience needle phobia, stop the acupuncture immediately, remove all needles, lie down flat, and keep warm. If there is slight subcutaneous bleeding causing a small bruise after the procedure, it usually resolves on its own without treatment. If the local swelling and pain are severe or the bruising covers a large area, apply cold compresses to stop the bleeding. For mild discomfort after the acupuncture, gently pat the area up and down with your fingers until it subsides or improves. For more severe cases, in addition to patting the area up and down, other treatments can be used under the guidance of a doctor. If you or your research doctor determines that you cannot tolerate these adverse reactions, you may be asked to withdraw from the study. In the event of any injury related to this study, appropriate treatment will be provided. If the acupuncturist is unable to manage the situation, a consultation and handling will be arranged by a specialist physician.

**Ⅵ. Other alternative treatments for the disease involved in the clinical trial**

You can choose not to participate in this study, which will have no adverse effect on your access to routine care. At present, the conventional treatment for your health condition is: diuretics (such as hydrochlorothiazide) and β receptor blockers Inhibitors (such as metoprolol), calcium channel blockers (such as amlodipine), angiotensin II antagonists (such as Losartan, etc.), angiotensin-converting enzyme inhibitors (such as captopril), etc., please take under the guidance of a doctor.

**Ⅶ. Costs associated with participating in the trial**

For patients participating in the trial: We will provide 12 free acupuncture treatments and two free head MRI scans (MRI scans cause no radiation or other harm). Any costs and compensation for adverse events related to the intervention during the trial will be handled according to relevant laws and regulations, with the research team bearing responsibilities. This study has no compensation for transportation expenses, lost work expenses, etc.

**ⅦI. Confidentiality of research**

All information concerning you, including your identity, medical history, condition, physical examination, and laboratory test results, will be strictly confidential within the bounds of the law. Investigators, monitors appointed by the sponsor, ethics committees, and national food and drug administration authorities are permitted to review your medical records related to this study to verify the authenticity and accuracy of the data collected. However, personal details will not be disclosed. Your name will not appear in any public materials or reports related to this study.

**Ⅸ.Clarify subjects' rights**

You participate in the study entirely on a voluntary basis. You have the right to withdraw from the clinical trial at any stage and your withdrawal will not result in any penalty or loss of benefit, nor will it affect your doctors treatment of you. If you decide not to participate in this study or wish to withdraw at any time after the study has begun, please contact your doctor promptly.

Your doctor may terminate this study without your consent in the following circumstances:

A Out of consideration for your treatment

B You failed to follow the relevant regulations of the study, did not take medicine on time according to the doctors instructions or did not carry out all examinations on time

C Study terminated

**X. Handling of complaints from subjects**

During the study, if you have any complaints about participating in the study, please contact the Medical Ethics Committee of Beijing University of Chinese Medicine at 010-539 11431.

**Informed consent form·Page of consent signature**

**Subject statement**

I have carefully read the "Informed Consent for Clinical Research Subjects" and fully understand the purpose, content, methods of this clinical study, as well as the potential benefits and risks of participating in the study. The doctor has provided clear explanations of relevant medical terms, and all my questions have been answered in simple terms. I understand that I can refuse to join the study or terminate and withdraw at any time and under any circumstances without affecting my medical treatment or rights.

My participation in this study is completely voluntary and has been fully considered. I have understood the therapeutic effects and possible risks of the study on my disease and obtained complete and true information related to this study. I fully understand and support this clinical study. Under no pressure and free choice, I volunteered to participate in this clinical study, and voluntarily cooperated with the research doctor to complete the clinical study according to the prescribed treatment and scale evaluation.

I agree to allow the project management department of the Ministry of Science and Technology to verify the case data when necessary.

I will receive a copy of an informed consent form signed and dated.

Subject (signature):

Date: Year Month Day

(Or legal representative (signature):) Relationship with the subject:

contact number:

**┄┄┄┄┄┄┄┄┄┄┄┄┄┄┄┄┄┄┄┄┄┄┄┄┄┄┄┄┄**

**Statement by the researchers**

I declare that I have explained the contents, procedures and possible risks and benefits of this study to the above participants in detail, and have given full answers to any questions raised by the subjects, who have received satisfactory answers and expressed understanding.

Researcher (signature):

Date: Year Month Day

contact number:
