# Supplementary material for: Efficacy and neural mechanism of acupuncture for essential hypertension: Study protocol for a randomized clinical trial
Source: PLoS One. 2025 Sep 19;20(9):e0332268. doi: 10.1371/journal.pone.0332268 (PMC12449014; doi:10.1371/journal.pone.0332268)
Supplement: S4 File — (DOCX) [file pone.0332268.s004.docx]

**Outline of clinical study protocol design**

**I. Research content**

Subjects: patients with essential hypertension (EH) and healthy controls (HC)

Intervention: acupuncture

Primary outcome: change in office systolic blood pressure at 4 weeks of treatment compared to baseline

Study design type: randomized controlled trial

**Ⅱ. Research plan**

1. Research subjects

1.1 Western medical diagnostic criteria

Referring to the diagnostic criteria for essential hypertension (EH) in the "The 2018 revision of the Guidelines for Hypertension Prevention and Treatment in China" revised by the Chinese Hypertension Revised Committee. In the absence of antihypertensive medication, three consecutive office blood pressure measurements (on different days) with systolic blood pressure (SBP) ≥140 mmHg and/or diastolic blood pressure (DBP) ≥90 mmHg. Hypertension is also diagnosed in people with a history of hypertension who are currently taking antihypertensive medications and whose blood pressure is lower than 140/90 mmHg.

1.2 Inclusion criteria

① Grade 1 hypertension, SBP between 140-159mmHg and between 90-99 mmHg;

② No antihypertensive drugs used;

③ Age: 25-60 years old, male or female, right-handed;

④ No language and intellectual disability, can answer and fill in the questionnaire smoothly;

⑤ Sign the informed consent.

1.3 Exclusion criteria

① Diseases affecting blood pressure, such as renal parenchymal disease, renal artery stenosis and other vascular diseases, obstructive sleep apnea syndrome, primary aldosteronism;

② Use of other drugs affecting blood pressure in the past month (except antihypertensive drugs), such as hormone drugs, central nervous system drugs, non-steroidal anti-inflammatory drugs, etc.;

③ Uncontrolled diabetes mellitus (glycated hemoglobin ≥ 6.5%);

④ Suspected or confirmed history of alcohol or drug abuse;

⑤ Women of childbearing age who are not using contraception, pregnant and lactating women;

⑥ Individuals carrying pacemakers, defibrillators, vascular clips, implantable electrical or magnetic devices, mechanical heart valves, cochlear implants, and other functional MRI contraindications, as well as those with claustrophobia; individuals with metal fragments in their face or eyes, working with metals; individuals found to have clear organic lesions or severe asymmetry of skull anatomy during MRI scanning; and individuals who cannot undergo fMRI for other reasons;

⑦ Received acupuncture treatment for hypertension in the past 3 months;

⑧ Patients who have participated in other clinical trials in the past month.

Those who meet one or more of the above requirements will be excluded.

1.4 Exit criteria

① Drop-out criteria: Subjects who have completed the course of treatment and observation period specified in this protocol (≤80% of the prescribed treatment) for some reason after informed consent and qualified for randomization are considered as drop-out cases.

② Handling of drop-out cases: When a subject drops out, the investigator should contact the subject as much as possible by visiting, making an appointment for follow-up, or calling to ask for reasons and complete the evaluation items. The relevant test data should be properly kept for both file and statistical processing of the full analysis set. Shedding patients do not need replacement.

1.5 Suspension standard

① Serius adverse events occur, and the clinical trial of this case should be stopped according to the judgment of the doctor;

② If other diseases or symptoms affecting the observation of the trial appear and the clinical trial should be stopped according to the doctors judgment, the case shall be treated as invalid.

③ Important deviations occurred in the implementation of clinical trial protocols, such as poor compliance, which made it difficult to evaluate the efficacy of acupuncture.

④ Subjects who are unwilling to continue the clinical trial and request to withdraw from the clinical trial during the clinical trial.

1.6 Exclusion criteria

① Those who meet the inclusion and exclusion criteria but cannot cooperate with treatment on time will be excluded.

② After the subjects were removed, the researcher should record the changes in the condition by telephone. The removal of patients does not require replacement.

2. Sample size estimation

The clinical part of this study was designed as a randomized controlled trial, with the primary outcome measure being the decrease in SBP at week 4 compared to baseline for sample size calculation. Based on the results of the pre-experiment, using PASS software, it was estimated that the difference in SBP change at week 4 between the electro-acupuncture group and the sham electro-acupuncture group should be 4±5 mmHg. Considering a 5% bilateral significance level and an 80% confidence level, each group required 26 patients. Taking into account a 20% dropout rate, each group needed to recruit 33 participants, totaling 66 participants across both groups.

3.Study grouping (randomization)

This study randomly divided EH patients into an electro-acupuncture group and a sham electro-acupuncture group at a ratio of 1:1, with each group consisting of 33 cases. The random sequence was generated by professional statisticians using Stata V.12.0 software. When qualified participants were available, a fixed person not involved in the study assigned the random numbers via telephone. Thirty-three healthy subjects were included, matched for age and gender with the patients. The healthy control group participants did not receive any treatment but were required to undergo a single MRI scan and complete a questionnaire during the study period.

1. Treatment plan

4.1 acupuncture treatment

① Acupuncture group

Acupoints: Quchi, Hegu, Zusanli, Taichong

Location:

Quchi: On the lateral aspect of the elbow, at the midpoint of the line connecting LU5 with the lateral epicondyle of the humerus.

Hegu: On the dorsum of the hand, radial to the midpoint of the second metacarpal bone.

Zusanli: 3 cun directly below Dubi (ST35), and one finger-breadth lateral to the anterior border of the tibia..

Taichong point: In the depression anterior to the junction of first and second metatarsal bones.

Acupoint location is referred to the national standard "Acupoint Name and Location" (GB/T12346-2021). Acupuncture operation refers to the operation method of the national traditional Chinese medicine college planning textbook "Acupuncture" in the new century. Specific positioning and operation methods are as follows:

Needle: Use disposable sterile acupuncture needle (0.30mm x 40mm)

Operate:

(1) Bilateral Taichong and Hegu points: The patient lies on his back, and after routine disinfection with 75% alcohol, the acupuncture physician applies needles through the skin 0.5-0 .8 inches, the application method is to connect 2hz electroneedle after obtaining qi, and the needle is left for 30 minutes.

(2) Other acupoints: Routine acupuncture was performed with reference to the planning textbook "Science of Acupuncture and Moxibustion" of the national colleges of traditional Chinese Medicine in the new century. Disposable sterile acupuncture needles were used. After injection, each point was injected for about 10s until qi was obtained, and the needles were left for 30 minutes.

② Control group

Points: Acupuncture physicians perform shallow puncture on the four non-acupoints listed in the table.

Needle: Use disposable sterile acupuncture needle (0.30mm x 40mm)

Operation: The patient was in a supine position, and the conventional disinfection was performed with 75% alcohol. The disposable sterile acupuncture needle (produced by Suzhou Huatuo Medical Equipment Co., LTD., 0.30mm x 40mm) will be used. The acupuncturist inserted the needle into the skin 4mm without Deqi, and no electricity was generated after connecting the 2hz electric needle. Leave the needle for 30 minutes.

| **Table 1 Non-acupoints** | |
| --- | --- |
| NA | Locations |
| NA 1 | 1 cun beside LR3, on the first metatarsal bone. |
| NA 2 | 1 cun back and outside of ST36. |
| NA 3 | In the middle of LI4 and LU10 (between the lung and large intestinal meridian). |
| NA 4 | In the middle of LI11 and LU5 (between the lung and large intestinal meridian). |

Treatment time: The above two groups were treated 3 times a week, and the needle was left for 30 min each time. It was advisable to treat every 1-2 days, and a total of 12 treatments were given in 4 weeks.

③ Healthy control group: The subjects in the healthy control group did not receive acupuncture treatment

4.2 Record medication

If the patient has poor effect of needle pricking to lower blood pressure after enrollment, the specialist doctor will consult and determine the plan to control blood pressure according to the condition, such as adjusting lifestyle, taking appropriate antihypertensive drugs and determining the appropriate dosage and type of medication, and truthfully record the information of the patients drug type, dosage and frequency in the later period.

1. Observation indicators and observation time points

5.1 Clinical efficacy indicators (primary and secondary outcomes)

Primary outcome measure: change in office SBP score at 4 weeks of treatment compared to baseline SBP will be measured according to the WHO stepwise approach, using an automatic sphygmomanometer (HEM-7136, Omron, Kyoto, Japan) after the patient has rested in a seated position for at least 5 minutes. Baseline measurements are taken from both arms, with the upper arm kept level with the heart, and the arm with higher blood pressure will be measured throughout the study. Blood pressure is repeated every 5 minutes for 3 times, and the average of the last two readings is taken. The change in systolic blood pressure SBP from baseline at week 4 is calculated as the primary outcome measure.

Secondary outcome:

① Change in SBP from baseline at other time points;

(Evaluation time points: Week 2, Week 8)

② Differences in DBP changes;

(Evaluation time points: baseline, week 2, week 4, and week 8)

③ Change in the health survey short form (SF-12) from baseline;

(Evaluation time points: baseline, week 4, week 8)

④ International Physical Activity Questionnaire

(Evaluation time points: baseline, week 2, week 4, week 8)

⑤ Pittsburgh Sleep Scale;

(Evaluation time points: baseline, week 4, week 8)

⑥ State-trait anxiety scale (STAI);

(Evaluation time points: baseline, week 2, week 4, and week 8)

⑦ The proportion of patients with good blood pressure control (blood pressure <140/90mm Hg);

(Evaluation time points: baseline, week 2, week 4, and week 8)

Other indicators

① Blind evaluation

(Evaluation time points: Week 2, Week 4)

5.2 MRI evaluation index

Two groups of patients who received intervention were scanned by functional magnetic resonance imaging (fMRI) before treatment and 4 weeks after intervention to evaluate the imaging mechanism of acupuncture. The first baseline image data of hypertensive patients were compared with those of healthy controls.

Brain function indicators (fMRI)

① Functional connectivity (FC) based on brain island seed points

Brain structural indicators (sMRI)

② Cortical thickness (CT) and cortical surface area (SA)

Cerebral blood flow index (FSL)

⑤ Local blood flow (rCBF)

5.3 Security indicators

Adverse events: hematoma, hypnosis, post-procedure discomfort, fMRI scan discomfort, etc.

Evaluation time: The evaluation was performed after each treatment or fMRI scan

Object of evaluation: each subject

Measures: If the subject has an adverse event, the researcher shall report it truthfully and record it, and give treatment according to the actual situation of the subject. If necessary, the trial may be suspended.

1. Efficacy evaluation criteria

According to the 2018 Revised Chinese Hypertension Prevention and Treatment Guidelines formulated by the Hypertension Alliance (China), the Chinese Medical Association of Cardiology Branch, and the Hypertension Professional Committee of the Chinese Medical Doctor Association, blood pressure is defined as: systolic blood pressure <140mmHg and diastolic blood pressure <90mmHg.

1. Adverse events

The adverse reactions were evaluated at any time during the acupuncture process, including skin bruising, needle fainting, needle stagnation, and acid swelling after the acupuncture treatment. Any adverse events that occur during the study period should be recorded in detail, and serious adverse events should be treated immediately and reported within 24 hours.

1. Data entry and statistical analysis

Statistical analysis was performed using SPSS v20.0 software. For continuous data, mean ± standard deviation (M±SD) or median and interquartile range were used, while for categorical data, frequency, proportion, and percentage were used. For comparisons of two groups of continuous data, independent samples t-tests or rank-sum tests were employed; for comparisons of two groups of categorical data, χ2 tests or rank-sum tests were used. The significance level was set at 0.05, meaning that P <0.05 indicates a statistically significant difference. For all randomized cases, intention-to-treat (intention-to-treat analysis, ITT) analysis was conducted, and missing data in case records were imputed using multiple imputation (Multiple imputation).

The DPARSF toolkit based on Matlab2016b and SPM12 was used to preprocess fMRI image data, with the DPABI V6.1 software package for functional connectivity analysis. Multiple comparisons were corrected using the Gaussian random field toolkit, with Cluster P-values set at 0.01 and Voxel P-values at 0.05. The "Recon-all" command in Freesurfer 6.0.0 software was used to segment, register, reconstruct, and parameterize 3D T1 data. Cortical thickness and cortical surface area were extracted from the corresponding ROI. Data statistical analysis was performed using DPABI and SPSS software. Mixed effects models were used for group comparisons, while paired t-tests were used for intra-group comparisons, with age, gender, education duration, and head movement parameters as covariates. Visualization was conducted using BrainNet Viewer and Graphpad prism software.

1. Quality control

① Subjects were included in strict accordance with the diagnostic, inclusion and exclusion criteria.

② After discussion by experts, relevant standard operating procedures (SOP) are formulated for each link of the experiment, so that there is a unified standard for the operation of each link and there is a basis to follow when opinions are inconsistent.

③ Researchers must undergo unified training. The content includes familiarizing themselves with the objectives and requirements of the study, mastering relevant diagnostic and therapeutic standards, acupuncture operation methods, and the use of evaluation forms. Different training durations should be set for researchers with different responsibilities. Researchers with the same responsibilities within the research center must pass a consistency test before conducting experiments.

④ Strict records and summaries of clinical trials. The center uses a uniformly printed case report form (CRF), which is uniformly numbered and registered for allocation. Operations are strictly carried out according to the project design plan, with CRF forms filled out carefully and objectively, and all issues arising during clinical trials truthfully recorded.

⑤ Enhance compliance control. Register the phone numbers and addresses of participants, maintain communication with them. Proactively contact participants when they fail to attend multiple sessions to understand their situation. Fully inform patients about the purpose and significance of the study, obtain informed consent, and ensure that acupuncture treatment costs and related examinations are free, funded by the research project.

⑥ When 10% and 90% of the subjects are included, the inspector shall record the records and data collection of the random test study book. If any problems occur, report them to the superior in time, find out the problems, solve the problems and strictly implement them.
