# Supplementary material for: Efficacy and neural mechanism of acupuncture for essential hypertension: Study protocol for a randomized clinical trial
Source: PLoS One. 2025 Sep 19;20(9):e0332268. doi: 10.1371/journal.pone.0332268 (PMC12449014; doi:10.1371/journal.pone.0332268)
Supplement: S5 File — (DOCX) [file pone.0332268.s005.docx]

**临床研究方案设计提纲**

**一、研究内容**

研究对象：原发性高血压患者及健康对照（HC）

施加措施：针刺

效应指标：治疗4周诊室收缩压较基线变化值

研究设计类型：随机对照试验

**二、研究方案**

（一）研究对象

1、西医诊断标准

参照中国高血压修订委员会所修订的《中国高血压防治指南2018年修订版》中原发性高血压（EH）的诊断标准。未使用降压药物的前提下，3次测量诊室血压（非同日），收缩压（SBP）≥140 mmHg和/或舒张压（DBP）≥90 mmHg。对既往有高血压病史，目前正服用降压药物，血压虽然低于140/90 mmHg者，也诊断为高血压。

2、纳入标准

①1级高血压，收缩压SBP在140-159 mmHg /舒张压 DBP在90-99 mmHg之间；

②未使用降压药；

③年龄25-60岁，男女不限，右手利；

④无语言及智力障碍，可顺利回答和填写问卷；

⑤签署知情同意书。

3、排除标准

①肾实质性疾病、肾动脉狭窄及其他血管病、阻塞性睡眠呼吸暂停综合征、原发性醛固酮增多症等影响血压的疾病；

②过去1个月使用影响血压的其他药物（除外降压药）：如激素类药物、中枢神经类药物、非甾体类抗炎药物等；

③未受控制的糖尿病（糖化血红蛋白≥6.5%）；

④怀疑或确有酒精、药物滥用病史；

⑤备孕、育龄期妇女未避孕者、妊娠期及哺乳期妇女；

⑥ 体内携带心脏起搏器、除颤器、血管夹、植入性电或磁装置、机械心脏瓣膜、人工耳蜗等 功能磁共振（fMRI ）禁忌物及幽闭恐惧症者；脸部或者眼睛内有金属碎片，从事金属相关工作；MRI 扫描中发现有明确器质性病变或严重头颅解剖结构不对称者；其他原因不能接受 fMRI 扫描者；

⑦ 近3月内因高血压病接受针灸治疗；

⑧ 近1个月参加其他临床试验的患者。

满足以上 1 项或多项要求者即予排除

5、退出标准

①脱落标准：经知情同意、并筛选合格进入随机化试验的受试者，因故未完成本方案所规定的疗程及观察周期（≤80%规定治疗），作为脱落病例。

②脱落病例的处理：当受试者脱落后，研究者应采取登门、预约随访、电话等方式，尽可能与受试者联系，询问理由，完善评估项目。脱落病例均应妥善保存有关试验资料，既作留档，也是进行全分析集统计处理所需。脱落病人无需另补。

6、中止标准

①出现严重的不良事件，根据医生的判断需停止该病例临床试验；

②出现其他影响试验观察的病证，根据医生判断应该停止临床试验者，作无效病例处理。

③临床试验方案实施中发生了重要偏差，如依从性太差等，难以评价针灸疗效。

④ 受试者在临床试验过程中不愿意继续进行临床试验，提出退出临床试验的要求者。

7、剔除标准

①符合纳入排除标准但不能按时配合治疗者予以剔除。

②当受试者剔除后，研究者应采取电话方式进行询问病情变化予以记录。剔除病人无需另补。

（二）样本量估算

本研究临床部分为随机对照试验设计，以第4周SBP较基线下降值为主要结局指标进行样本量计算。根据前期预试验结果，使用 PASS 软件估算，假设电针组第4周SBP变化值较假电针组下降差值为4±5mmHg，考虑5%双侧显著性水平和80%把握度，需每组患者26人。考虑 20%的脱落率，每组需要招募33人，两组共计66人。

1. 研究分组（随机方法）

本研究按1:1的比例将EH患者随机分为电针组和假电针组，每组各33例。随机序列由专业的统计人员应用 Stata V.12.0 软件编程产生，当有合格的受试者时，由一个不参与本研究的固定人员采用电话方式分配随机号。纳入与患者年龄性别相匹配的健康受试者33例，健康对照组受试者不参与治疗，仅需在研究期间进行一次磁共振扫描及问卷调查。

1. 治疗方案
2. **针刺治疗**

**①针刺组**

**取穴：**曲池、合谷、足三里、太冲

**定位：**曲池：在肘外侧，尺泽和肱骨外上髁连线的中点处。

合谷：在手背，第一掌骨和第二掌骨之间，约平第2掌骨桡侧的中点。足三里：在小腿外侧，犊鼻下3寸，犊鼻与解溪连线上。

太冲穴：在足背，第1、2跖骨间，跖骨底结合部前方凹陷中，或触及动脉搏动。

腧穴定位参照国家标准《腧穴名称与定位》（GB/T12346-2021）。针刺操作参考新世纪全国中医药院校规划教材《针灸学》的操作方法。具体定位和操作方法如下：

**针具：**使用一次性无菌针灸针（0.30mm×40mm）

**操作：**

1. 双侧太冲穴和合谷穴：患者仰卧位，使用 75%酒精常规消毒后，针灸医师施针穿透皮肤0.5-0.8寸，施用手法以得气后连接2hz电针，留针30分钟。
2. 其余腧穴：参考新世纪全国中医药院校规划教材《针灸学》行

常规针刺，采用一次性无菌针灸针，进针后各穴行针约10s至得气，留针30分钟。

**②对照组**

**取穴：**针灸医师按表中的4个非穴进行浅刺。

针具：使用一次性无菌针灸针（0.30mm×40mm）

**操作：**患者仰卧位，使用 75%酒精常规消毒后，使用一次性无菌针灸针（苏州华佗医疗器械有限公司生产，0.30mm×40mm），针灸医师施针刺入皮肤4mm，不得气，连接2hz电针后不通电；留针30分钟。

| **表1 非穴定位** | |
| --- | --- |
| 非穴 | 定位 |
| 非穴1 | 尺泽与曲池连线中点（肺经和大肠经之间）太冲旁开1寸，第一趾跖骨面上 |
| 非穴2 | 合谷与鱼际连线中点（肺经和大肠经之间） |
| 非穴3 | 足三里后外侧1寸 |
| 非穴4 | 太冲旁开1寸，第一趾跖骨面上 |

**治疗时间：** 以上两组每周治疗 3 次，每次治疗留针 30 min，治疗以间隔 1-2 天为宜，4 周共计 12 次治疗。

**③健康对照组：**健康对照组受试者不进行针刺治疗

1. **记录用药**

若入组后患者出现针刺降压效果不佳的情况，请专科医生会诊并结合病情确定控制血压的方案，例如调整生活方式、服用适当的降压药并确定合适的服药剂量和种类，并如实记录后期患者服药种类、剂量、频率信息。

1. **观察指标及观察时点**

**1、临床疗效指标（主要和次要疗效指标）**

**主要结局指标：治疗4周诊室收缩压（SBP）评分较基线变化值**

SBP将按照世卫组织的步骤方案进行测量，并每次使用自动测量仪（HEM-7136, Omron, Kyoto, Japan），患者静坐休息至少5 min后测量。基线时测量两臂血压，保持上臂与心脏水平，血压较高的手臂将在整个研究过程中进行测量。每5 min重复测量血压3次，取最后2次平均值。计算受试者第4周收缩压SBP较基线变化值，作为主要结局指标。

**次要结局指标：**

①其他时间点收缩压（SBP）较基线变化值；

（评价时间点：第 2 周、第8周）

②舒张压（DBP）变化的差异；

（评价时间点：基线、第 2 周、第 4 周、第8周）

③ 健康调查简表（SF-12）较基线变化值；

（评价时间点：基线、第 4 周、第8周）

④ 国际体力活动问卷

（评价时间点：基线、第 2 周、第 4 周、第8周）

⑤ 匹兹堡睡眠量表;

（评价时间点：基线、第 4 周、第8周）

⑥状态-特质焦虑量表 (STAI);

（评价时间点：基线、第 2 周、第 4 周、第8周）

⑦血压控制良好（血压<140/90mm Hg)的患者比例；

（评价时间点：基线、第 2 周、第 4 周、第8周）

**其他指标**

①盲法评价

（评价时间点：第 2 周、第 4 周）

1. **影像评价指标**

两组接受干预的患者在治疗前和干预4周后各进行1 次磁共振成像扫描，进行针刺影像学机制评价。高血压患者第一次基线影像数据与健康对照组进行对比分析。

**脑功能指标（fMRI）**

①基于脑岛种子点的功能连接（FC）

**脑结构指标（sMRI）**

②皮层厚度(CT)和皮层表面积（SA）

**脑血流指标（FSL）**

③局部血流 (rCBF)

1. **安全性指标**

不良事件：血肿、晕针、针刺后遗感、fMRI扫描不适等情况

评价时点：受试者每次治疗或fMRI扫描结束后均进行评价

评价对象：每个受试者

处理措施：受试者出现不良事件，研究人员需如实上报记录，并根据受试者实际情况给予治疗处理，必要时可中止试验。

**（六）疗效评价标准**

参照高血压联盟（中国）、中华医学会心血管病学分会、中国医师协会高血压专业委员会制定的《中国高血压防治指南2018年修订版》，血压达标的定义为：收缩压＜140mmHg且舒张压＜90mmHg。

**（七）不良事件**

针刺过程中随时进行不良反应评价包括皮肤淤青、晕针、滞针、

针刺治疗结束后的酸胀感等情况。对于研究期间出现的任何不良事件

作详细记录，严重不良事件需立即处理并在 24 小时内上报。

**（八）数据录入与统计分析**

用 SPSS 软件统计分析，计量资料采用均数±标准差（M±SD或中位数和四分位数间距表示，计数资料采用频数、构成比、百分比表示。计量资料的两组比较，采用独立样本的 t 检验或秩和检验，计数资料的两组比较采用χ2 检验或秩和检验。检验水准为 0.05，即 P ＜0.05 被认为所检验的差别有统计学意义。对所有经随机化分组的全部病例，采用意向性分析（intention-to-treat analysis, ITT），对有缺失数据的病例资料，采用多重插补法（Multiple imputation）填补。

采用基于Matlab2016b和SPM12的DPARSF工具包对fMRI图像数据进行预处理，DPABI V6.1软件包计算功能连接。使用高斯随机场工具包进行多重比较校正，Cluster的P值取0.01，Voxel的P值取0.05，使用Freesurfer 6.0.0软件的“Recon-all”命令对3D T1数据进行行分割、配准、三维重建及参数计算。提取相应ROI的皮层厚度和皮层表面积等数据。利用DPABI和SPSS软件进行数据统计分析。采用混合效应模型进行组间比较，配对样本t检验进行组内比较，将年龄，性别，教育年限和头部运动参数作为协变量。采用BrainNet Viewer、Graphpad prism软件进行可视化。

**（九）质量控制**

①　严格按照诊断、纳入和排除标准进行受试者的纳入。

②　经专家讨论，试验各个环节制定相关标准操作流程（SOP），使得各环节的操作有统一的标准，意见不一致时有依据可循。

③　研究人员必须经过统一培训。内容包括熟悉本研究的目标、要求，掌握有关诊疗标准、针刺操作方法、测评表的使用等，并对不同分工的研究者设立不同的培训时长，研究中心的相同分工的研究者需要通过一致性检验才能开展试验。

④　严格临床试验的记录及总结。中心采用统一印制的病例报告表（CRF），统一编号并登记分配情况。严格按照课题设计方案进行操作，认真、客观地填写CRF表，如实记录临床试验中出现的各种问题。

⑤　加强依从性控制。登记受试者的电话和地址，与受试者保持沟通。在其多次未治疗时主动联系受试者，了解其相关情况。充分让病人了解研究的目的和意义，签署知情同意书，针灸治疗费用及相关检查免费，由课题经费支出。

⑥　当纳入10%及90%的受试者时，监察员进行随机检测研究记录本的记录情况及数据采集情况。如遇问题及时向上级反映，发现问题、解决问题并严格执行。
