# Supplementary material for: Efficacy and neural mechanism of acupuncture for essential hypertension: Study protocol for a randomized clinical trial
Source: PLoS One. 2025 Sep 19;20(9):e0332268. doi: 10.1371/journal.pone.0332268 (PMC12449014; doi:10.1371/journal.pone.0332268)
Supplement: S6 File — (DOCX) [file pone.0332268.s006.docx]

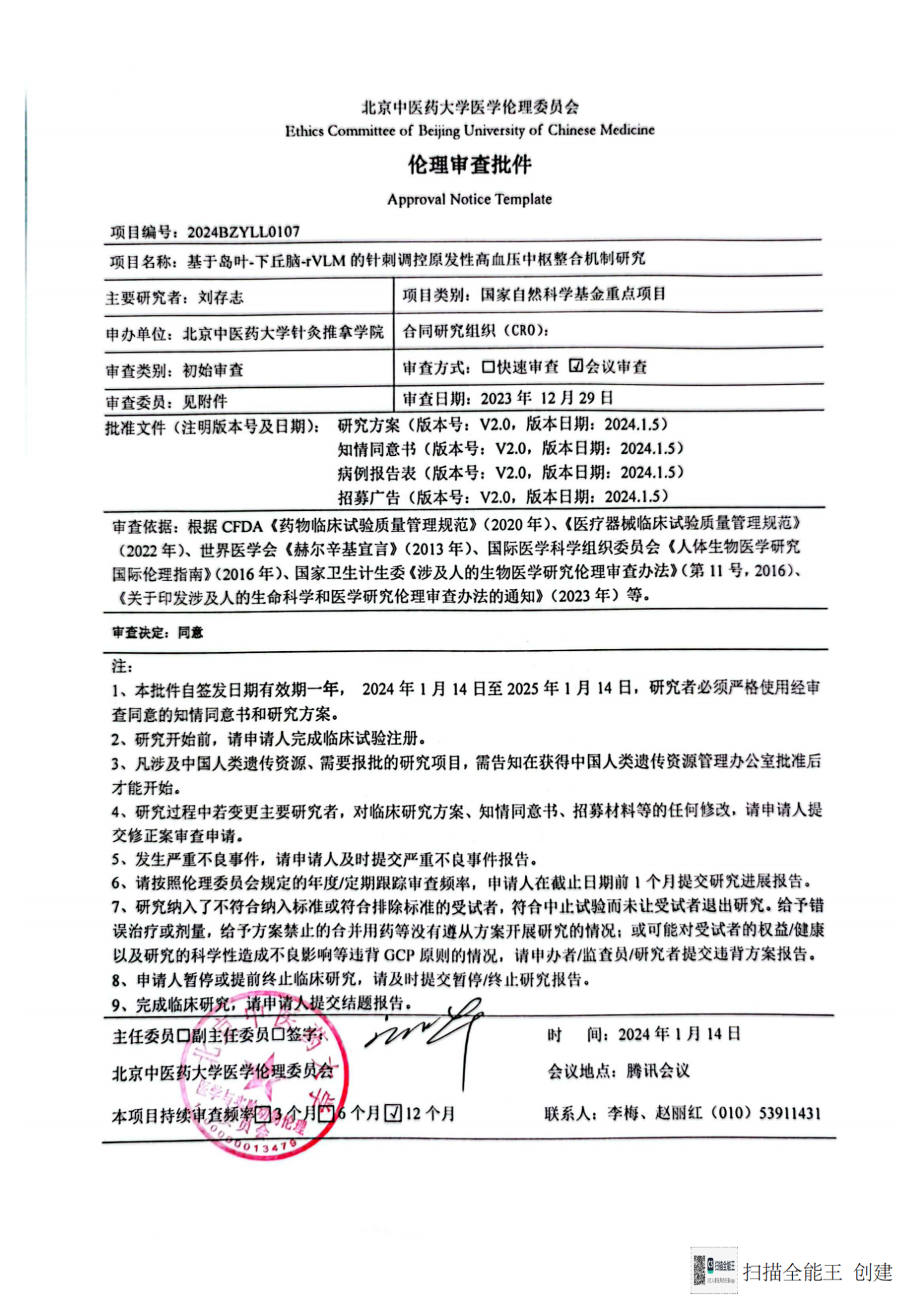


Ethics committee of Beijing University of Chinese Medicine

Approval Notice Template

| Project Number：2024BZYLL0107 | |
| --- | --- |
| Project Title：Neuroimaging study on the central integration mechanism of acupuncture regulation in essential hypertension based on insula- hypothalamus-rVLM | |
| Principal Investigator：Cun-Zhi Liu | Project Type：Key Project of National Natural Science Foundation of China |
| Bid unit：School of Acupuncture-Moxibustion and Tuina, Beijing University of Chinese Medicine | Contract Research Organization： |
| Review Type：Initial review | Review Mode：Meeting review |
| Review committee：attached | Review Date：December 29, 2023 |
| Approval Document：Study protocol, version: 2.0; version date: January 05, 2024  Informed consent form, version: 2.0; version date: January 05, 2024  Case Report Form, version: 2.0; version date: January 05, 2024  Recruitment advertisement, version: 2.0; version date: January 05, 2024 | |
| The Basis of Review：  “Good Clinical Practice for Drugs” (2020) and “ Good Clinical Practice for Medical Devices” (2022) issued by CFDA, “Declaration of Helsinki” (2013) issued by World Medical Association, “International Ethical Guidelines for Health-related Research Involving Humans” (2016) issued by Council for International Organizations of Medical Sciences, “Methods of ethical review of biomedical research involving human beings” (2016) and “Notice on the issuance of ethical review procedures for life sciences and medical research involving humans” (2023) issued by National Health and Family Planning Commission of the People's Republic of China, etc. | |
| Review decision：Approved | |
| Note:  1. This approval notice template is valid for one years from the date of issue, i.e. from 14 January 2024 to 14 January 2025. The researcher must strictly use the informed consent form and study protocol approved by the review.  2. Before the study begins, the applicant is required to complete the registration for clinical trials.  3. Any study project involving human genetic resources in China that needs to be submitted for approval must be informed that it can only be started after obtaining approval from Human Genetic Resources Administration of China (HGRAC)  4. During the trial, if there are changes on the principal investigator, the study protocol, informed consent, recruitment materials, etc., the applicant is required to submit an amendment review application.  5. If a serious adverse event occurs, the applicant must submit a serious adverse event report in time.  6. According to the annual/periodic follow-up review frequency prescribed by the ethics committee, the applicant must submit a research progress report 1 month before the deadline.  7.In the following cases, the sponsor/ monitor/ researcher is required to submit a Protocol Deviation Report: Subjects who did not meet the inclusion criteria or meet the exclusion criteria were included；Subjects who meet suspension criteria were not withdrawn from the study; The study was not performed in accordance with the protocol, such as giving wrong treatment or dosage, or concomitant medications prohibited by the protocol, etc.; The study may violate GCP principles, such as adversely affecting the rights/health of the subject and the scientific nature of the research.  8. If the project has been suspended or stopped in advance, the applicant is required to submit the Suspension/ Termination Report in time.  9. If the project has been completed, the applicant is required to submit the Final Report. | |
|  |  |
|  |  |
| Chairman：Jian-Ping Liu Date：January 14, 2024  Ethics committee of Beijing University of Chinese Medicine Review Site：Tencent Meeting  Frequency of periodical review：12 months Contacts：Mei Li, Li-Hong Zhao 010-53911431 | |
|  |  |
|  |  |
